# Saturated fatty acids negatively affect musculoskeletal tissues *in vitro* and *in vivo*

**DOI:** 10.1016/j.mbplus.2024.100153

**Published:** 2024-05-31

**Authors:** Ryan T. Lin, Benjamin Osipov, Danielle Steffen, Marin Chamberlin, Suraj J. Pathak, Blaine A. Christiansen, Kevin J.M. Paulussen, Keith Baar

**Affiliations:** a Department of Neurobiology, Physiology & Behavior, University of California Davis, 1 Shields Avenue, 195 Briggs Hall, Davis, CA 95616, USA; b University of Pittsburgh School of Medicine, 3550 Terrace St, Pittsburgh, PA, USA^1^; c Department of Orthopaedic Surgery, University of California Davis Health, Sacramento, CA, USA; d Physiology and Membrane Biology, University of California Davis, Davis, CA, USA

**Keywords:** Diet, Exercise, Tendon, Muscle, Bone, Palmitate

## Abstract

Fish oils rank among the world’s most popular nutritional supplements and are purported to have numerous health benefits. Previous work suggested that fish oils increase collagen production; however, the effect of fish oils on musculoskeletal health is poorly understood. Further, the divergent effects of omega-3 (Ω3FA) and saturated fatty acids (SFA) remains poorly understood. We tested the effects of Ω3FA and SFAs on *in vitro*-engineered human ligament (EHL) function. EHLs were treated with bovine serum albumin (BSA)-conjugated eicosapentaenoic acid (EPA, 20:5(n-3)), palmitic acid (PA, 16:0), or a BSA control for 6 days. EPA did not significantly alter, whereas PA significantly decreased EHL function and collagen content. To determine whether this was an *in vitro* artifact, mice were fed a control or high-lard diet for 14 weeks and musculoskeletal mass, insulin sensitivity, and the collagen content, and mechanics of tendon and bone were determined. Body weight was 40 % higher on a HFD, but muscle, tendon, and bone mass did not keep up with body weight resulting in relative losses in muscle mass, tendon, and bone collagen, as well as mechanical properties. Importantly, we show that PA acutely decreases collagen synthesis *in vitro* to a similar extent as the decrease in collagen content with chronic treatment. These data suggest that Ω3FAs have a limited effect on EHLs, whereas SFA exert a negative effect on collagen synthesis resulting in smaller and weaker musculoskeletal tissues both *in vitro* and *in vivo*.

## Highlights

• The saturated fatty acid palmitate deceased engineered human ligament mechanics and collagen content.
• A 14-week high fat diet decreased the collagen content of the Achilles tendon resulting in a decrease in relative stength.
• A high fat diet similarly decreased bone collagen content and bone mineral density and increased trabecular spacing.
• *In vitro,* palmitic acid decreased procollagen synthesis in the first twenty-four hours after feeding.
• A high fat diet decreases collagen synthesis in musculoskeletal tissues which may contribute to increased fracture and tendon/ligament injury rates.

## Introduction

The musculoskeletal system is composed of tissues (bone, muscle, tendon, ligament, and cartilage) whose primary purpose is to provide structural support and allow movement. Since musculoskeletal function is integral to human movement, it should not be surprising that the annual direct cost of musculoskeletal injuries in the United States is in excess of $250 billion dollars, more than diabetes and heart disease combined [1].

Connective tissues like bone, tendon, ligaments, and cartilage are collagen rich tissues that were traditionally viewed as inert [2], [3], [4]. However, this notion has been questioned by more recent research that shows these tissues are responsive to both exercise and diet [5], [6], [7], [8]. These studies suggest that nutrition can act synergistically or antagonistically with activity to improve or impair collagen synthesis and musculoskeletal function [7], [9], [10].

Omega-3 fatty acids (Ω3FA) are essential fatty acids that must be obtained through diet. Ω3FA have been extensively studied in the context of collagen synthesis for inflammatory disorders and dermatology [11], [12], [13]. In 2000, Hankenson and colleagues demonstrated that the omega-3 eicosapentaenoic acid (EPA, 20:5(n-3)) significantly increased collagen production in pig medial collateral ligament (MCL) cells [14]. Where EPA may increase collagen synthesis, saturated fatty acids (SFAs) have been suggested to be toxic to cultured ligament cells [15]. In fact, Takeuchi and colleagues have shown that palmitic acid (PA; 16:0) can drive apoptosis in periodontal ligament cells [16]. Together, these data suggest that FAs may have divergent effects on connective tissue collagen production and by extension musculoskeletal health.

To date, the effects of EPA and PA have only been tested in 2D culture. The problems with 2D cell culture are that treatments tend to last a few hours and only surrogate measures (cell number, collagen mRNA, etc.) of connective tissue function are possible. Since connective tissues serve a mechanical role in the body, being able to determine the mechanical changes resulting from an intervention is essential. For example, as a tendon/ligament develops, cell number decreases. Therefore, measures of apoptosis of ligament cells in 2D could either indicate that the treatment is killing the cells, or that the treatment is increasing the maturity of the tissue. By contrast, 3D engineered ligaments can be treated *in vitro* for days to weeks and at the end of treatment the tissues can be mechanically tested to determine how the intervention altered tissue function [17]. Engineered human ligaments (EHLs) are a widely used model for the effect of exercise [18], hormones [19], [20], and nutrition [21] on ligament mechanics. However, the effect of FA supplementation has not been addressed in this model [19], [22], [23], [24]. Further, whether the mechanical effects identified using EHLs translate to *in vivo* models remains an open question.

The aim of this study was to evaluate the effect of fatty acids, specifically the omega-3 fatty acid EPA and the saturated fatty acid PA, on EHLs. Since these data showed a negative effect of PA on ligament function, we then determined whether a high saturated fat diet in mice could have the same effect on musculoskeletal tissues *in vivo*. Our primary hypothesis was that EPA would improve, and PA would impair collagen synthesis and mechanics both *in vitro* and *in vivo*.

## Results

### Effect of fatty acids on EHL mechanics and collagen content

**Fig. 1:**
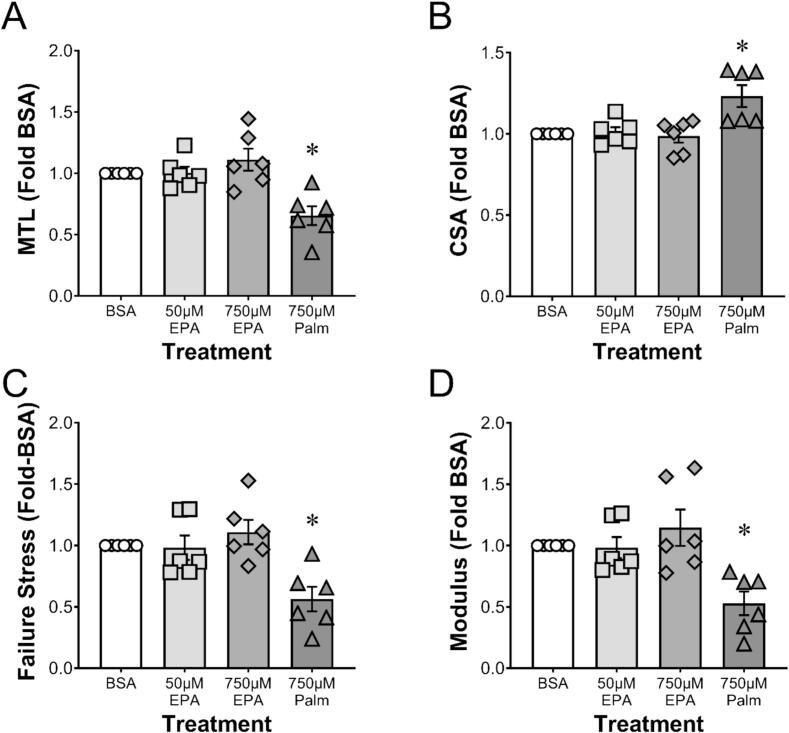
(A) Maximal tensile load (MTL), (B) cross-sectional area (CSA), (C) failure stress, and (D) modulus as a function of fatty acid level. Data are for bovine serum albumin (BSA), low-dose EPA (50 µM EPA), high-dose EPA (750 µM EPA), and PA (750 µM PALM). Each point represents a different experiment with n = 3–8 technical replicates per group where mean data were made relative to BSA and then plotted as a group. * indicates significant difference (p < 0.05) compared to all other groups.

Treatment of engineered ligaments with 50 or 750 µM EPA had no effect on EHL mechanical or material properties. By contrast, 750 µM PA decreased the maximal tensile load (MTL), failure stress, and modulus compared to all other groups (Fig. 1A, C, D).

**Fig. 2:**
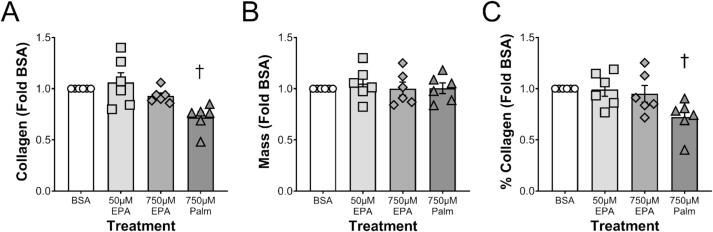
(A) Collagen, (B) mass, and (C) % collagen as a function of fatty acid level. Data are for BSA, low-dose EPA (50 µM EPA), high-dose EPA (750 µM EPA), and PA (750 µM PALM). Each point represents a different experiment with n = 3–8 technical replicates per group where mean data were made relative to BSA and then plotted as a group. † indicates significant difference (p < 0.05) compared to BSA.

The collagen data mirrored the mechanical data. Neither 50 nor 750 µM of EPA had any effect on collagen content or concentration in the EHLs. Again, 750 µM PA had a negative effect on the collagen content and the percent of the tissue dry mass that was collagen compared to the BSA control group (Fig. 2A, C).

### Body mass, muscle mass, and insulin sensitivity of muscle

**Fig. 3:**
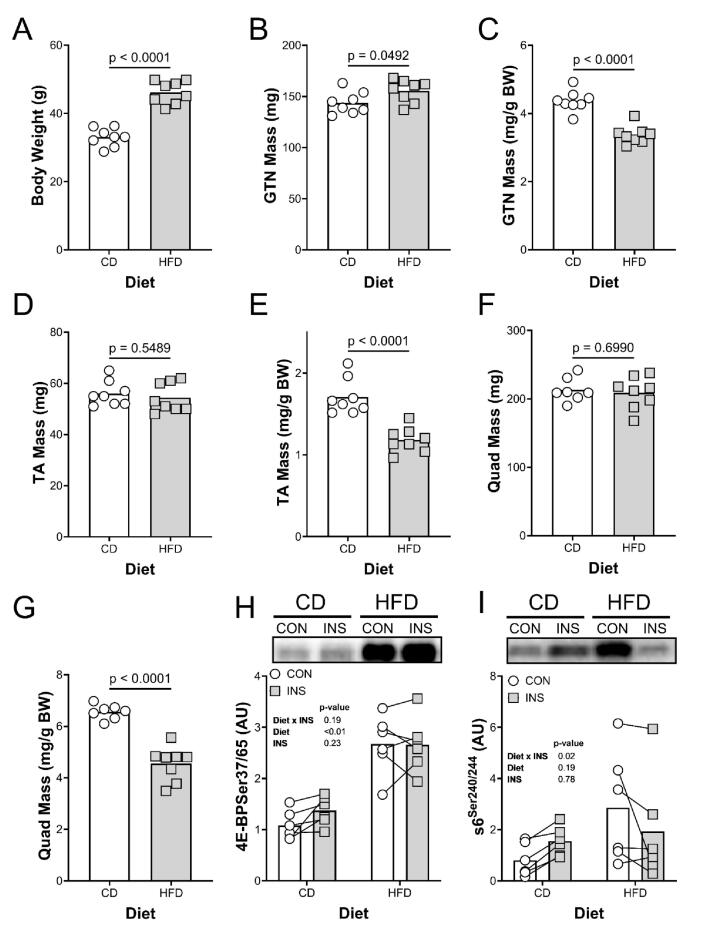
(A) Body weight, (B) gastrocnemius (GTN) muscle mass, (C) GTN mass/body mass, (D) quadriceps mass, (E) quadriceps mass/BW (F) tibialis anterior (TA) mass, and (G) TA mass/body mass for control (CD, n = 8) and high fat diet (HFD, n = 8) mice. To gauge muscle insulin resistance, soleus muscles were incubated in CON or INS stimulation media for 10 min and (H) 4E-BP, and (I) s6 phosphorylation were measured. All stats in the figure. N = 5–8 per group/treatment.

As expected, a 14-week high saturated fat diet increased body weight compared to the control diet fed mice (Fig. 3A). The absolute gastrocnemius (GTN) muscle mass was significantly greater in the HFD mice relative to the CD (Fig. 3B). By contrast, the absolute mass of neither the tibialis anterior (TA; Fig. 3D) nor quadriceps (Quad; Fig. 3F) muscles were different than mice on a CD. Relative to body mass, the GTN, TA, and QUAD muscles were all significantly smaller in the HFD mice (Fig. 3C, E, G).

To determine whether the HFD decreased muscle insulin sensitivity, the response to insulin and amino acids was determined in the soleus muscle. As expected, there was an increase in baseline mechanistic target of rapamycin complex (mTORC)1 activity as determined by measuring 4E-BP1 and s6 phosphorylation as a function of diet (Fig. 3H, I). Insulin treatment increased phosphorylation in all the CD fed muscles, whereas insulin treatment did not change or decreased phosphorylation in the muscles from HFD-fed mice.

### Achilles tendon mechanics and collagen

**Fig. 4:**
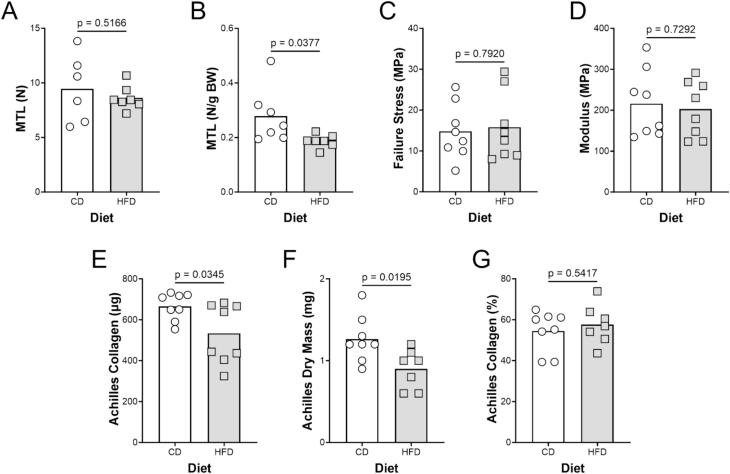
(A) MTL, (B) MTL/Body mass, (C) failure stress, and (D) modulus for control (CON, n = 8) and high fat diet (HFD, n = 8) mice. (E) Collagen, (F) dry mass, and (G) % collagen. All mice (n = 8) are shown on each graph along with the statistical measures.

Absolute MTL was not different in the HFD mice relative to those on the CD (Fig. 4A). However, normalizing the MTL to body mass, we found a significant decrease in the relative strength of the Achilles (Fig. 4B). Neither the failure stress nor the modulus was different as a function of diet (Fig. 4C, D).

The Achilles tendon was significantly smaller (lower mass) in animals on HFD (Fig. 4F). The decrease in dry mass was mirrored by a decrease in total collagen within the tendon (Fig. 4E). However, the percent collagen was not different from control (Fig. 4G).

### Bone mass, and collagen content

**Fig. 5:**
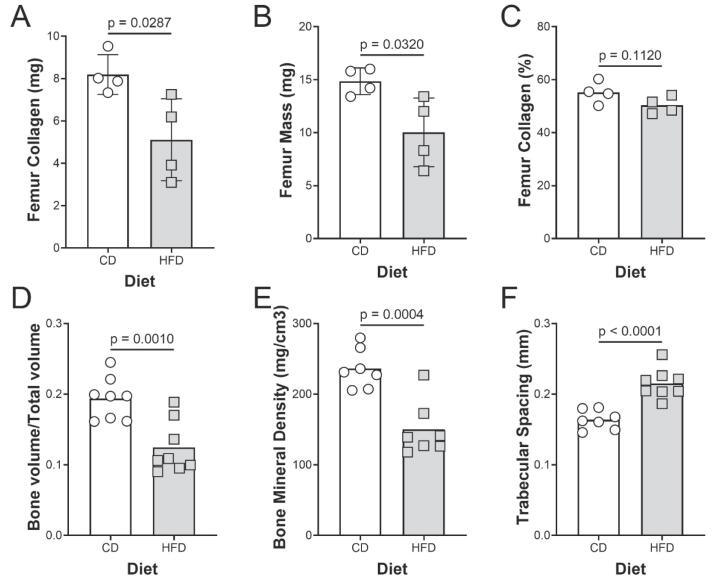
Bone collagen and structural properties for mice on HFD. (A) Collagen content, (B) dry mass, and (C) collagen concentration of the femurs of control (CON, n = 4) and high fat diet (HFD, n = 4) fed mice. (D) Bone volume/total volume, (E) bone mineral density, and (F) trabecular spacing (n = 8).

As with the Achilles tendons, the collagen content and dry mass of the femurs from HFD fed mice were significantly lower than those of the CD fed animals (Fig. 5A, B). Again, like the Achilles, the HFD did not change the percent collagen within the tissue (Fig. 5C). However, the decrease in bone collagen content was associated with a decrease in bone mass, decrease in bone volume fraction, bone mineral density, and an increase in trabecular spacing (Fig. 5 D, E).

**Fig. 6:**
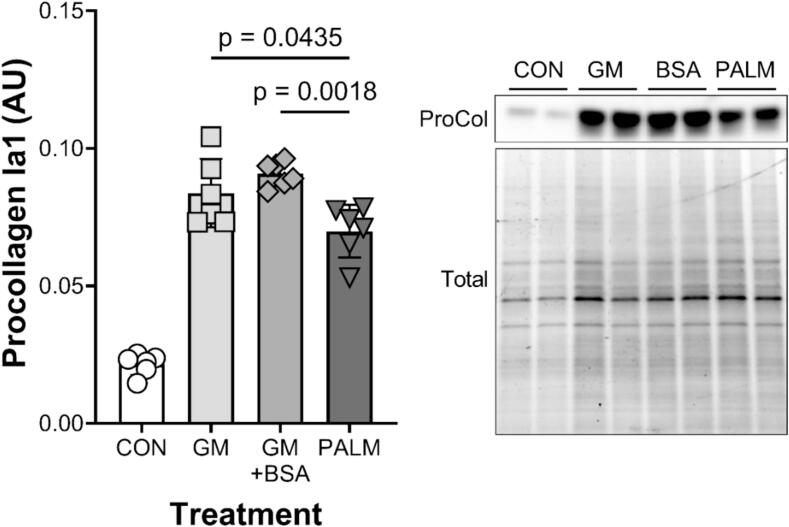
Procollagen Ia1 levels in human ACL fibroblasts treated with PA. Procollagen Ia1 protein content in human ACL cells treated with growth media (GM), growth media + BSA, or 750 µM palmitate (PALM) for 24 h. Each point represents a technical replicate with n = 6 per group and the bar represents the mean value per group. Procollagen Ia1 levels in the graph relative to the total protein in the lane (measured using a stain-free gel, see methods). P-values from the ANOVA performed are indicated for the PALM group relative to GM or GM + BSA.

To begin to understand how saturated fatty acids were negatively affecting the collagen content of tissues, we determined procollagen production in response to a single feed with and without palmitic acid *in vitro*. As can be seen in Fig. 6, the normal increase in procollagen levels seen 24 h after a feeding (growth media (GM); control media (GM + BSA)) was significantly inhibited by ∼25 % by 750 µM palmitate (PALM).

## Discussion

This study investigated the effect of fatty acids or a high fat diet on musculoskeletal collagen content and function. Contrary to our initial hypothesis that EPA would improve collagen content and mechanics, EPA had no effect on the collagen content or mechanical properties of the EHLs. Even though EPA had little effect on EHLs, the primary saturated fatty acid in humans (palmitic acid; PA) significantly decreased EHL mechanics and collagen content. Further, using a high saturated fat diet to test the effect of high saturated fatty acid levels in rodents, we found that a 14-week HFD increased body weight, decreased relative muscle mass and insulin sensitivity. Interestingly, the HFD did not induce changes in the absolute strength of the Achilles even though the animals weighed 40 % more and the absolute GTN mass was 8 % greater. This means that relative to body and muscle weight Achilles strength decreased. Since muscle strength relative to body weight is a good predictor of performance [25], data from the current work reflect a significant decrease in functional performance. A similar decrease in bone quantity was observed on HFD. In both the Achilles tendon and the femur, collagen content and tissue dry mass were lower in HFD fed mice. To begin to understand why collagen content is lower in the presence of high saturated fatty acids, we treated human ACL fibroblasts with PA for 24 h *in vitro* and measured and impaired accumulation of procollagen Ia1. Together, these data suggest that saturated fatty acids decrease collagen synthesis, which over time can impair musculoskeletal function.

The lack of effect of EPA on EHL mechanics or collagen content does not support our primary hypothesis that Ω3FA would increase collagen content and improve ligament mechanics. This hypothesis was based on the fact that in 2D cell culture Hankenson and colleagues found that Ω3FA increased collagen synthesis 13 % in pig medial collateral ligament cells [14]. However, this group did not determine the rate of matrix degradation or the incorporation of collagen into the extracellular matrix. The same group found that EPA had a similar effect on collagen synthesis in 3T3 mouse fibroblasts [26]. However, the effect of EPA in these studies is difficult to understand since, in the same work, lipopolysaccharide (LPS) increased collagen production, even though inflammation is generally recognized to decrease collagen in connective tissues [27], [28], [29], and we have shown that inflammation decreases collagen content and mechanics in EHLs [30]. Moving to a 3D model allowed us to treat for a longer period (6 days versus 2 days) and test the mechanical properties and accurately determine the amount of collagen within the extracellular matrix. With these advancements, we were able to refute the idea that EPA could induce long-term changes in collagen turnover or tissue mechanics. This finding is consistent with Ezure and Amano who saw no difference in either cell abundance or *ColIa1* expression compared to control as a function of EPA [31]. These data suggest that even though EPA may have anti-inflammatory effects in the cardiovascular system [32], [33], [34] and in patients with cancer [35], the effect of EPA on the musculoskeletal system is less clear [36], [37].

In contrast to EPA, PA had a significant adverse effect on EHLs, decreasing mechanical properties ∼40 % and material properties (failure stress and modulus) by more than 50 %. In the six independent experiments treating EHLs with fatty acids presented in Fig. 1, PA decreased mechanical and material properties in each, whereas there was no effect of EPA. Similarly, PA decreased collagen content and concentration by approximately 28 % compared to the BSA control. The negative effect of PA on connective tissue *in vitro* has been previously reported. Ezure and colleagues found that PA at concentrations of 10 µM were sufficient to significantly decrease human dermal fibroblast cell abundance and *ColIa1* expression compared to control [31]. PA has also been found to induce apoptosis [15], [16] and promote hyperinflammation [38], [39] in human periodontal ligament fibroblasts. By contrast, the current study suggests that the negative effects of PA on collagen production are seen within 24 h of treatment; a time when total cellular protein is not negatively affected by the saturated fatty acid. These data suggest that PA impairs collagen synthesis even without altering cell number.

The negative effect of PA on EHLs was more severe than that seen *in vivo* with a high fat diet. *In vivo*, the effects of high saturated fatty acids were smaller. First, we confirmed that the HFD was effective in reproducing the muscular effects reported previously, including insulin resistance and sarcobesity [40], [41]: the relative loss of skeletal muscle mass resulting from obesity. Here, the GTN, which as a load-bearing muscle had to support the 45 % increase in body mass, increased absolute mass by only 8 %, whereas the positional TA muscle and the QUAD muscle showed no difference in absolute mass. The result was that relative to body mass all muscles were significantly smaller than those of the control diet fed mice. Together with the decrease in relative muscle mass, baseline mTORC1 signaling was higher in the soleus muscles of mice on a HFD and the stimulatory effect of insulin was lost. This is consistent with the loss of insulin sensitivity seen in mice on a HFD reported previously [40], [42], [43]. Together, these data show that the HFD effectively induced insulin resistance in the musculoskeletal system.

Negative effects of a HFD on bone were also observed *in vivo*. In the bone, we have confirmed previous studies showing that HFD-fed mice have reduced rates of bone collagen synthesis compared to controls [44], [45], [46]. Furthermore, the effects of HFD on mouse bone microarchitecture observed in this study are similar to what has been previously reported [47], [48], [49]. The finding of lower collagen content in both Achilles tendons and femurs of mice suggests that collagen turnover in both tendon and bone are negatively impacted by an HFD. Our data suggests that impaired collagen synthesis is the primary driver of the loss of musculoskeletal function since acute PA treatment decreased procollagen Ia1 production ∼25 % (Fig. 6) and this corresponds well with the decrease in collagen content of the EHLs (∼28 %) after 6 days of treatment. Previous studies have linked Runx2 [50] and Osterix [51] as possible transcriptional regulators of bone collagen synthesis that are negatively affected by HFD. By reinforcing the previously known effects of HFD on collagen synthesis in tendon and ligament as well, our data indicates that a HFD impairs collagen production across all musculoskeletal tissues.

Specific research into the negative effects of a high fat diet on tendon or ligament *in vivo* is scarce. Mooney and colleagues found that mice on a high fat diet demonstrated increased osteoarthritis and cartilage injury compared to lean control mice [10]. However, whether this was a function of the metabolic environment or excess weight is difficult to delineate. Previous studies that tested the effect of a high fat diet on tendon or ligament mechanics *in vivo* have shown similarities to the present study. Rios and colleagues found that tail tendons from rats who ate a HFD for 12 weeks had no significant differences in mechanical or biomechanical properties [52]. However, longer feeding protocols have found that the biomechanical properties of rodent tendons differentiate from controls anywhere between 15 weeks [53] and 48 weeks [54] on a HFD. These studies have shown that rodents who were fed a HFD over a prolonged period had increased body weight [53], [54], [55], [56], decreased MTL [53], [54], decreased failure stress [53], and decreased modulus [55], [56]. Thus, a decrease in tendon mechanical and material properties is a consistent finding in rodents on a HFD. Our finding that PA acutely decreases collagen synthesis suggests that impaired collagen synthesis leads to impaired biomechanical properties *in vivo*.

It is important to note that cells from all tissue donors responded similarly to the addition of PA. This is not surprising since we have previously shown that there is greater difference between replicates from the same donor than there is between donors [19]. Further, we have seen few differences between EHLs engineered in a standard hormone mixture from cells isolated from women or men [19].

There were a number of important limitations to the current work that must be acknowledged. First, we did not determine the effect of Ω3FAs on musculoskeletal function *in vivo*. Despite the fact that EPA had no effect on EHLs, it may have altered the physiological milieu *in vivo* resulting in augmented collagen synthesis. Second, the *in vitro* work was performed on ligament cells and engineered human ligaments and the *in vivo* work looked at tendon and bone function. We have found no difference *in vitro* between tissues engineered from ligaments (ACL, MCL, or PCL) or tendon (hamstring, quad, or Achilles) cells; however, whether the EHL data directly translates to *in vivo* ligament data has not been demonstrated. Another possible limitation is that the *in vitro* work was performed in human cells, whereas the *in vivo* work was performed in mice. We used the EHLs to humanize the work as much as possible; however, whether the musculoskeletal response to HFD would be the same in people needs further substantiation.

In conclusion, we have shown that palmitic acid significantly decreased collagen synthesis *in vitro* and this resulted in impaired engineered human ligament mechanics. Further, a similar effect, lower collagen content and mechanics was observed in all musculoskeletal tissues examined *in vivo* after feeding a high saturated fat diet. By contrast, the Ω3FA EPA did not significantly affect the mechanics or collagen content of EHLs but we did not determine the effect of this macronutrient *in vivo*. Since the EHLs mimic the negative effect of a high fat diet *in vivo*, these engineered tissues may provide a unique approach to more thoroughly investigate the effect of saturated fatty acids on musculoskeletal tissue mechanics and develop interventions to mitigate these effects.

## Experimental procedures

### Cell isolation and culture

Human anterior cruciate ligament (ACL) cells were collected during ACL surgical reconstruction following informed consent, and all procedures and experiments were approved by the University of California Davis Institutional Review Board (IRB No. 779755). Donors included a 21-year-old female and a 24-year-old male. Both donors had no significant prior medical history and required surgery secondary to trauma. The tissue remnants were incubated with 1 mg/mL collagenase type II dissolved in growth media (GM; Dulbecco’s Modified Eagles Medium (DMEM) containing 10 % fetal bovine serum and 0.1 % penicillin) for 17 h at 37 °C. Cells were isolated away from the collagen, plated, and passaged at less than 70 % confluence twice before freezing cells for subsequent experiments. To begin an experiment, a vial of cells was thawed, and the cells expanded for two passages. In passage four, 25 plates were used to make cohorts of 24 EHLs.

### Engineered human ligament formation and culture

Brushite anchors were prepared by mixing powdered β-tricalcium phosphate and 3.5 M orthophosphoric acid in a 1:1 μL:mg ratio. This mixture was added to 3D printed teardrop molds with pins and centrifuged at 2250 times gravity at 4 °C for 1 min. Anchors were allowed to set overnight before being removed from the molds and pinned 12 mm apart on 35 mm plates that had been lined with 1.5 mL of polydimethylsiloxane (PDMS; Sylgard, Dow, Wilmington, DE). The plates were sterilized with two treatments of 70 % ethanol before 2.5 × 10^5^ cultured ACL fibroblasts were plated within a fibrin gel containing 714 μL thrombin, 286 µL of 20 mg/ml fibrinogen, 20 µg aprotinin, and 2 µg amino hexanoic acid. To allow fibrin clot formation, the EHLs were incubated at 37 °C with 5 % CO_2_ for 15 min. Each EHL was then fed 2 mL GM containing 200 μm ascorbic acid, 50 μm proline, and 5 ng/mL of transforming growth factor-β1 (TGF-β1). On day 8, treatment-specific feeding began until the ligaments were tensile-tested on day 14 [24].

### EHL treatment

The EHL experiments tested the effect of bovine serum albumin (BSA) conjugated EPA at 50 or 750 µM, 750 µM PA, or the BSA vehicle control. The fatty acid dosing was selected based on the work from the Hundal group on the differential effects of palmitate and palmitoleate on muscle cells [57]. In the aforementioned studies, the effect of the PA reached a maximum by 750 µM and therefore this level was used for the *in vitro* assays for the current work. Fatty acids were initially suspended in ethanol and then sterile filtered. On the day of feeding, each FA was added to growth media containing 10 % volume per volume BSA and then incubated at 37 °C for 1 h to promote conjugation. Ethanol alone was added to the GM-10 %BSA mixture for an hour to create the control media. Ascorbic acid, proline and TGF-β1 were added to all treatments in the same concentrations as GM [21].

We treated the EHLs between 8 and 14 days in culture since at this time point the collagen content and material properties of the tissues are increasing exponentially [17]. Interventions that decrease collagen synthesis or incorporation into the tissue have a dramatic effect on function and collagen content at 14 days [17]. This may overestimate the effect *in vivo* but provides high power for identifying interventions that affect tendon/ligament development and function.

### EHL mechanical testing

Mechanical testing of EHLs was performed on day 14 following formation. The length and width of EHLs were measured using digital calipers. Graft depth was defined as 0.5 mm for the calculation of cross-sectional area (CSA). Constructs were then placed into 3-D printed teardrop grip molds and pulled until failure using a custom-built tensile tester. A LabView program (National Instrument) used to load the EHLs to failure without preconditioning at a rate of 0.4 mm/s. Maximal force before failure was used as the maximal tensile load (MTL) Failure stress was calculated by dividing MTL by CSA and Young’s modulus (stiffness) was calculated as the maximal slope of the stress–strain graph.

### EHL collagen measurement

After tensile testing, samples were dried and weighed before being hydrolyzed in 6 N HCl at 120 °C for 30 min. The lids of the tubes were opened and the HCl evaporated in a fume hood at 120 °C for 90 min. The resulting pellet or an increasing amount of pure hydroxyproline (Sigma, St. Louis, MO) was suspended in 200 µL hydroxyproline buffer containing 570 mM sodium hydroxide, 588 mM sodium acetate, 173 mM citric acid, and 140 mM acetic acid. An aliquot of each sample was diluted with 150 µL chloramine T solution and incubated for 20 min, before addition of 150 µL aldehyde-perchloric acid with 60 % 1-propanol, 5.8 % perchloric acid, and 1 M 4-(Dimethylamino)benzaldehyde. The samples were incubated for 15 min at 60 °C. Absorbance of 200 µL of the solution was read at 550 nm on an Epoch Microplate Spectrophotometer (BioTek Instruments Ltd, Winooski, VT, USA). Collagen mass was calculated relative to the hydroxyproline standard curve assuming collagen contains 13.7 % hydroxyproline and % collagen was calculated by dividing the collagen mass by the dry mass of the EHL [20].

### Mice and diets

All animal experiments adhered to and were approved by the University of California Davis Institutional Animal Care and Use Committee under Protocol 22957. For this experiment, eight C57BL/6J DIO male control mice and eight C57BL/6J male diet-induced obesity mice (The Jackson Laboratory, Sacramento, CA) were housed in cages with a 12-h light and dark cycle and *ad* libitum access to food and water. Mice started the diet at 6 weeks of age. Control (CD) mice were fed Research Diets, Inc. D12450B (10 kcal% fat) and high-fat diet (HFD) mice were fed Research Diets, Inc. D12492 (60 kcal% fat). Diets were closely matched for fiber content (CD = 50 mg/g; HFD = 65 mg/g), whereas cholesterol and other animal fats are ∼10-fold higher in the HFD. Mice were aged 20 weeks when tissues were collected for testing (14 weeks on diet).

### Tissue collection and insulin sensitivity

At the time of sacrifice, body weight of mice was obtained, the animals were anesthetized using isoflurane and both soleus muscles were carefully removed so that the Achilles was left intact to determine insulin sensitivity. Briefly, once removed the soleus muscles were pinned at resting length on cork and placed muscle side down in Krebs-Henseleit Buffer (KHB) at 37 °C with constant rotation. After 10 min of pre-incubation, one of the muscles was moved to a control solution (KHB) and the other placed in KHB containing 10µU/ml human insulin (Novolin, Novo Nordisk, Plainsboro, NJ) and 50 µM amino acids (50X MEM Amino Acids and 100X Glutamax, GIBCO, Waltham, MA) for 10 min before rinsing and freezing the muscle in liquid nitrogen. Muscles were stored at −80 °C until processed for western blotting.

During the soleus muscle incubations, the gastrocnemius (GTN) muscle-Achilles tendon and attached foot were removed, wrapped in PBS-soaked gauze, and stored in 50 mL tubes until mechanical testing. The femur was similarly removed, stripped of overlying muscle, and wrapped in PBS-soaked gauze before processing.

### Mechanical testing of the Achilles tendon

The mechanical properties of control (n = 8) and HFD (n = 8) mouse Achilles tendons (PT) were tested using a Model 68SC-1 single column tensile tester containing a 100 N load cell with a BioPuls Body Temperature Bath and Submersible Pneumatic Side Action Grips (Instron, Norwood, MA, USA). The length and width of the left and right Achilles tendons were measured with digital calipers. Length of the Achilles was measured from the base of the GTN to the calcaneus. CSA was calculated by measuring the width at the narrowest point using digital calipers with the assumption that the tendon had rectangular shape with a depth of 0.5 mm. All muscles were wrapped in 14 AWG (American wire gauge) wire before gripping to prevent lateral spreading within the grip. The muscle–tendon-bone unit was loaded onto the tensile tester by gripping the GTN muscle proximally and foot distally. Grip tension was held at 90 lb/in^2^ (56245.57 kg/m^2^).

Samples were immersed in PBS within the testing chamber bath at 37 °C. All tendons underwent 10 cycles of preconditioning (0.10–0.25 N, 0.25 mm/s) and then were elongated at 0.25 mm/s until force dropped by 80 %. MTL in Newtons was the maximal load measured prior to failure. Failure stress was calculated by normalizing the MTL by CSA. Young's modulus was calculated as the maximal slope of the stress–strain (displacement divided by the initial length) curve.

### Bone collagen content

Following removal of soft tissue, femurs from the right leg of half the mice were weighed before incubating in 0.5 M HCl for 18 h on a rocker at room temperature to demineralize the bone. Demineralized femurs were dried at 120 °C for 30 min and dry mass was measured. The femurs were then hydrolyzed in 6 N HCl at 120 °C for two hours, and then processed for hydroxyproline as described above.

### Bone MicroCT

Following removal of soft tissue, femurs from the left leg were preserved in 70 % ethanol. Bones were then scanned using micro-computed tomography (SCANCO Medical, μCT 35, Brüttisellen, Switzerland) with 6 mm nominal voxel size (X-ray tube potential = 55 kVp, current = 114 mA, integration time = 900 ms, number of projections = 1000/180°) according to the JBMR guidelines for µCT analysis of rodent bone structure [58]. All analyses were performed using the manufacturer’s analysis software. Trabecular bone was analyzed at the distal femoral metaphysis. Regions of interest were manually drawn on transverse images excluding the cortical surface beginning at the metaphyseal growth plate and extended 250 slices proximally (1500 µm). Trabecular bone volume fraction (BV/TV), trabecular thickness (Tb.Th), trabecular number (Tb.N), trabecular spacing (Tb.Sp), apparent bone mineral density (BMD), and bone tissue mineral density (TMD) were determined using the manufacturer’s analysis software.

### *In vitro* collagen synthesis assay

Primary human ACL fibroblasts were plated (2.5 x 10^5^ per well) and grown to confluence. Two days after reaching confluence, the cells were either left in existing media or fed 1 ml of growth media (DMEM containing 10 % fetal bovine serum and 1 % penicillin), the GM-10 %BSA control, or GM containing 750 µM BSA-conjugated palmitate. None of the media contained ascorbic acid so that procollagen Ia1 could not be secreted from the cell, allowing us to determine how much procollagen had been made since treatment. Twenty-four hours after treatment, the wells were aspirated, washed twice in ice-cold PBS, and collected for western blot. Samples (10 µL) were loaded onto a 4–20 % Criterion TGX Stain‐Free Precast Gel and run for 45 min at a constant voltage of 200 V. The gel was then activated under UV-light to quantify total protein within each well (Fig. 6). The total protein within the lane was used to normalize procollagen Ia1 to total protein. Proteins were then transferred to an activated PVDF membrane for 30 min at a constant voltage of 100 V. Transfer was confirmed by Ponceau staining the membranes. Membranes were then rinsed and blocked in 1 % fish skin gelatin in Tris‐buffered saline w/ 0.1 % Tween (TBST) for 30 min, washed in TBST, and then incubated at 4 °C overnight with the procollagen Ia1 primary antibody (SP1.D8 from the Iowa Hybridoma Bank; AB_528438) diluted in TBST at 1:1000. The following day, membranes were washed 3 times for 5 min with TBST and then incubated for 1 h at room temperature in a 0.5 % skim milk in TBST solution containing peroxidase‐conjugated secondary antibodies diluted at 1:10,000. Immobilon Western Chemiluminescent HRP substrate (Millipore, Hayward, CA, USA) was then applied to the membranes to visualize the proteins by chemiluminescence. Images were taken using the ChemiDoc MP System and bands were quantified using Image Lab (Bio‐Rad).

### Statistics

Statistical analysis was performed using GraphPad Prism, version 9.5.1 (San Diego, CA, USA). Group differences for the EHLs were compared with one-way ANOVA to test for statistical significance. When results reached statistical significance, post hoc analysis was performed using Tukey’s range test since all groups demonstrated equal variance. For the diet experiments, muscle, tendon, and bone samples were analyzed by *t*-test. A technical replicate was a single EHL, bone tendon or muscle within a group at a given time. Biological replicates reflect that the EHL experiments were repeated using either a new donor or a separate vial of cells from the same donor. Fold BSA was calculated for technical replicates by dividing the mean of each treatment group by the mean of the BSA control group to allow for comparison between experiments. Data is presented as mean and all replicates are shown on the graphs. A threshold of *p* < 0.05 set for statistical significance.

### CRediT authorship contribution statement

**Ryan T. Lin:** Writing – original draft, Formal analysis, Data curation, Conceptualization. **Benjamin Osipov:** Writing – review & editing, Investigation, Formal analysis, Data curation. **Danielle Steffen:** Writing – review & editing, Formal analysis, Data curation. **Marin Chamberlin:** Writing – review & editing, Methodology, Formal analysis, Data curation. **Suraj J. Pathak:** Writing – review & editing, Formal analysis, Data curation. **Blaine A. Christiansen:** Writing – review & editing, Supervision, Resources, Funding acquisition. **Kevin J.M. Paulussen:** Data curation, Formal analysis, Investigation, Writing – review & editing. **Keith Baar:** Writing – review & editing, Writing – original draft, Supervision, Resources, Project administration, Methodology, Investigation, Funding acquisition, Data curation, Conceptualization.

## Declaration of competing interest

The authors declare the following financial interests/personal relationships which may be considered as potential competing interests: Keith Baar reports financial support was provided by NIA. K. Baar has also received grants, consulting fees, speaking honoraria, and donations from nutritional companies such as PepsiCo, Bergstrom Nutrition, Ynsect, and GelTor to study the effect of dietary collagen on endogenous collagen synthesis.

## Data Availability

Data will be made available on request.
